# Elucidating the chemical profile and biological studies of *Verbascum diversifolium* Hochst. extracts

**DOI:** 10.3389/fphar.2024.1333865

**Published:** 2024-01-30

**Authors:** Sakina Yagi, Nilofar Nilofar, Abdullahi Ibrahim Uba, Giovanni Caprioli, Ahmed M. Mustafa, Simone Angeloni, Ismail Koyuncu, Fatma Seker, Rıdvan Polat, Sumaiya Jahan Supti, Faria Tasnim, Yusra Al Dhaheri, Gokhan Zengin, Ali H. Eid

**Affiliations:** ^1^ Department of Botany, Faculty of Science, University of Khartoum, Khartoum, Sudan; ^2^ Université de Lorraine, INRAE, LAE, Nancy, France; ^3^ Physiology and Biochemistry Research Laborotory, Department of Biology, Science Faculty, Selcuk University, Konya, Türkiye; ^4^ Department of Pharmacy, Botanic Garden “Giardino dei Semplici” “Gabriele d’Annunzio” University, Chieti, Italy; ^5^ Department of Molecular Biology and Genetics, Istanbul AREL University, Istanbul, Türkiye; ^6^ School of Pharmacy, University of Camerino, Camerino, Italy; ^7^ Department of Medical Biochemistry, Faculty of Medicine, Harran University, Sanliurfa, Türkiye; ^8^ Department of Biology, Science Arts Faculty, Harran University, Sanliurfa, Türkiye; ^9^ Department of Landscape Architecture, Faculty of Agriculture, Bingol University, Bingöl, Türkiye; ^10^ Department of Genetic Engineering and Biotechnology, University of Rajshahi, Rajshahi, Bangladesh; ^11^ Department of Biology, College of Science, United Arab Emirates University, Al-Ain, United Arab Emirates; ^12^ Department of Basic Medical Sciences, College of Medicine, QU Health, Doha, Qatar

**Keywords:** cancer, cardiovascular disease, Herbal medicine, oxidative stress, reactive oxgen species (ROS)

## Abstract

The present study was designed to evaluate the chemical composition, antioxidant, enzyme inhibition and cytotoxic properties of different extracts from aerial parts of *V. diversifolium* (family Scrophulariaceae), a plant that is native to Lebanon, Syria and Turkey. Six extracts, namely, hexane, dichloromethane (DCM), ethyl acetate (EtOAc), ethanol (EtOH), 70% EtOH, and water (aqueous) were prepared by maceration. The EtOH extract was predominated by the presence of rutin (4280.20 μg g^−1^) and *p*-coumaric acid (3044.01 μg g^−1^) while the highest accumulation of kaempferol-3-glucoside (1537.38 μg g^−1^), caffeic acid (130.13 μg g^−1^) and 4-hydroxy benzoic acid (465.93 μg g^−1^) was recorded in the 70% EtOH, aqueous, and EtOAc extracts, respectively. The EtOH (46.86 mg TE/g) and 70% EtOH (46.33 mg TE/g) extracts displayed the highest DPPH radical scavenging result. Both these extracts, along with the aqueous one, exerted the highest ABTS radical scavenging result (73.03–73.56 mg TE/g). The EtOH and 70% EtOH extracts revealed the most potent anti-AChE (2.66 and 2.64 mg GALAE/g) and anti-glucosidase (1.07 and 1.09 mmol ACAE/g) activities. The aqueous extract was the most efficacious in inhibiting the proliferation of prostate cancer (DU-145) cells with an IC_50_ of 8.71 μg/mL and a Selectivity Index of 3.7. In conclusion, this study appraised the use of *V. diversifolium* aerial parts as a potential therapeutic source for future development of phytopharmaceuticals that target specific oxidative stress-linked diseases including diabetes, cancer, cardiovascular disease, and Alzheimer’s disease among others.

## 1 Introduction

Oxidative stress, particularly reactive oxygen species (ROS), is a major contributor to the pathogenesis of several diseases (Shaito et al., 2022; Aramouni et al., 2023). Oxidative stress could mediate most chronic diseases by activating several transcription factors that can lead to the expression of many genes like those associated to inflammatory cytokines, chemokines, cell cycle regulatory molecules and anti-inflammatory molecules (Reuter et al., 2010). Living organisms employ well-controlled antioxidant defense systems to mitigate damages instigated by ROS (Badran et al., 2020). Many risk factors like aging or unhealthy lifestyles abate the effectiveness of these protective systems, and hence increase ROS accumulation, further precipitating or accentuating pathologies like Alzheimer’s, skin pigmentation, cardiovascular disease, diabetes, and cancer among others (Mesmar et al., 2021; Slika et al., 2022; Kıran et al., 2023).

Despite the tremendous efforts invested to prevent or treat these diseases, the overall incidence and associated morbidity remain alarmingly high (Kim et al., 2018). For instance, estimates suggest that over 45 million people are already affected by Alzheimer’s disease worldwide and this is expected to double every 20 years until 2050 (Santos et al., 2018). Diabetes mellitus is another disease with a rise of about 3% deaths between the year 2000–2019 and it was estimated that more than 1.31 billion people could be living with diabetes by 2050 worldwide (WHO, 2023a; Lancet, 2023). Similarly, cancer remained the second leading cause of global mortality, accounting for an estimated 19.3 million new cases and almost 10.0 million deaths in 2020 (Sung et al., 2021). Therefore, there is an ever-increasing need for concerted efforts to explore new targets and identify new molecules possessing a therapeutic value. One resource that is bountiful in such compounds is plants. Indeed, natural products from medicinal plants and their constituents serve as a crucial source of bioactives that appear promising to prevent or manage various diseases (Anwar et al., 2016; Anwar et al., 2017; Anwar et al., 2018; Kim et al., 2018; Samaha et al., 2019; Fardoun et al., 2020; AlKahlout et al., 2022). For example, three plants belonging to the genus *Verbascum* (family Scrophulariaceae), namely, *V. cheiranthifolium V. myriocarpum* and *V. pyroliforme*, are rich in phenolics and possess significant antioxidant and enzyme inhibitory activities (Zengin et al., 2023). Metabolites like verbascoside and chlorogenic acid in many *Verbascum* species activate endogenous antioxidant systems to defend against and scavenge free radicals, and hence ameliorate oxidative damage (Bender et al., 2021; Zengin et al., 2023).

*Verbascum diversifolium* (Synonyms: *V. mesopotamicum* Boiss.; *V. nitidulum* Freyn & Sint.) is a biennial plant that grows mainly in the temperate biome and mainly native to Lebanon, Syria and Turkey. Morphologically, *V. diversifolium* is characterized by deep tap roots, long stalked glandular hairs indumentum, lanceolate to linear-lanceolate, crenate-dentate basal leaves, cyme inflorescence (clusters of 1-4 flowers), triangular-lanceolate acutish calyx lobes, broadly ovate to subglobose capsules, as well as brown, oblong, and reticulate seeds (Karavelioğullari et al., 2011). In Turkish and Romanian traditional medicine, *V. diversifolium* is used to manage cough, inflammation including laryngitis, swelling, scars, and wounds, as well as an emollient or expectorant (Tita et al., 2009; Cakilcioglu et al., 2011). Chemically, *Verbascum* plants are characterized by the presence of iridoid and neolignan type glycosides, oleanan type terpenes, flavonoids, polysaccharides, saponins, steroids and alkaloids (Küçük et al., 2016; Blanco-Salas et al., 2021; Zengin et al., 2021; Zengin et al., 2023). They possessed versatile biological properties such as antioxidant, cytotoxic, antimicrobial and anti-inflammatory activities (Tatli and Akdemir, 2006a; Kahraman et al., 2012; Blanco-Salas et al., 2021; Donn et al., 2023). Moreover, bioactives of *Verbascum* species, including apigenin, verbascoside and chlorogenic acid were found to possess antioxidant, anticancer, anti-Alzheimer activities (Bender et al., 2021; Zengin et al., 2023). Despite the medicinal use of *V. diversifolium*, the literature is devoid of reports describing its chemical constituents or biological activity and hence, it was hypothesized that *V. diversifolium* could have a promising pharmacological potential like other species from this genus. The present study was undertaken to evaluate the chemical composition, antioxidant, enzyme inhibition and cytotoxic properties of different extracts from the aerial parts of *V. diversifolium*.

## 2 Materials and methods

### 2.1 Plant material

*V. diversifolium* plant materials were obtained from a field investigation (Kurudere Village, Bingol Turkey). Taxonomical identification was carried out by Dr. Rıdvan Polat and a specimen was deposited in the herbarium of Bingol University (Voucher Number: RP-1067). The aerial parts were carefully separated and dried in shade at room temperature, then ground into powder using a laboratory mill and stored in the dark.

### 2.2 Extraction

The extracts were prepared using the six solvents n-hexane, dicholoromethane, ethyl acetate, ethanol, ethanol/water (70%) and water. Each plant material (10 g) was macerated with 200 mL of the respective solvent at room temperature overnight. For water extract, the plant material (10 g) was soaked in boiled water for 15 min (as infusion method). The organic solvents were evaporated to remove solvents. The water extract was dried by a freeze drier.

### 2.3 Assay for total phenolic and flavonoid contents

The total content of phenols and flavonoids were assessed following the procedures described in the paper (Slinkard and Singleton, 1977).

### 2.4 HPLC-ESI-MS/MS

HPLC-MS/MS studies were performed using an Agilent 1290 Infinity series and a Triple Quadrupole 6420 from Agilent Technology (Santa Clara, CA) equipped with an electrospray ionization (ESI) source operating in negative and positive ionization modes. All details are given in Supplementary Material.

### 2.5 Antioxidant tests

*In vitro* antioxidant assays were conducted using the previously reported methods (Grochowski et al., 2017). For the 2,2-diphenyl-1-picrylhydrazyl (DPPH), 2,2′-azino-bis(3-ethylbenzothiazoline-6-sulfonic acid) (ABTS) radical scavenging, Cupric reducing antioxidant capacity (CUPRAC), and ferric reducing antioxidant power (FRAP) tests, the results were expressed as mg of Trolox equivalents (TE) per g extract. In the phosphomolybdenum (PBD) assay, the antioxidant potential was quantified in terms of mmol of Trolox equivalents (TE) per g extract. Metal chelating activity (MCA) was expressed as mg of disodium edetate equivalents (EDTAE) per g extract (Grochowski et al., 2017).

### 2.6 Enzyme inhibitory tests

The enzyme inhibition experiments for the samples were conducted following previously established protocols (Grochowski et al., 2017). Inhibition of amylase and glucosidase activities were expressed as mmol of acarbose equivalents (ACAE) per g extract, while inhibition of acetylcholinesterase (AChE) and butyrylcholinesterase (BChE) activities were expressed as mg of galanthamine equivalents (GALAE) per g extract. Tyrosinase inhibition was quantified as mg of kojic acid equivalents (KAE) per g of the tested extracts.

### 2.7 Cell analysis

#### 2.7.1 Materials

The following materials were used in this study: Cell culture medium (DMEM-F12/RPMI 1640; Sigma-Aldrich Cat No: D0697/R8758, United States), fetal bovine serum (FBS; Sigma-Aldrich Cat No: F7524, United States), %1 penicillin/streptomycin (Sigma-Aldrich Cat No: P4333, United States), L-glutamine (Sigma-Aldrich Cat No: 59202C, United States), trypsin-EDTA solution (Sigma-Aldrich Cat No: 59417C, United States), dimethyl sulfoxide (DMSO) (Sigma-Aldrich Cat No: PHR1309, United States), MTT (3-(4,5-Dimethylthiazol-2-yl)-2,5-Diphenyltetrazolium Bromide; Sigma-Aldrich Cat No: M2128, United States). Culture plates (96 well) were purchased from Nunc (Brand products, Denmark).

#### 2.7.2 Cell culture

Cancer and normal cell lines were obtained from ATCC and stored in liquid nitrogen: DU-145 (Prostate Carcinoma), MDA-MB-231 (Breast Adenocarcinoma), HELA (Cervix Adenocarcinoma), HT-29 (Colon Adenocarcinoma), HGC-27 (Gastric Cancer), and HEK-293 (Normal Human Embryonic Kidney) cells. These cells were cultured in DMEM-F12/RPMI-1640 media supplemented with 10% FBS and 100 μg/mL of streptomycin/100 IU/mL of penicillin in incubators at 37 °C under humid conditions containing 5% CO_2_.

#### 2.7.3 Cell viability assay

The cytotoxic effects of *V. diversifolium* (ethanol, ethanol/water and water) were assessed using the MTT (3-(4,5-Dimethylthiazol-2-yl)-2,5-Diphenyltetrazolium Bromide) assay. Cells (DU-145, MDA-MB-231, HELA, HT-29, HGC-27, and HEK-293) were seeded at 1 × 10^4^ cells per well in a 96-well sterile plate and incubated for 24 h. The media were then replaced with extracts at varying doses (0, 2.5, 5, 10, 25, 50, 100, and 200 μg/mL) for another 24 h. Subsequently, 10 μL of MTT solution (0.5 mg/mL) was added to each well as a reaction agent, followed by a 4-h incubation period. After removing the media, 100 μL of DMSO was added, and measurements were taken at OD570-OD690 nm using a plate reader (Thermo Multiskan GO, Thermo, United States). Finally, plots were generated, and the IC50 value was calculated.

#### 2.7.4 KEGG pathway analysis for CDK6 gene

KEGG (Kyoto Encyclopedia of Genes and Genomes) pathway analysis is a valuable approach in bioinformatics and systems biology for make sense of complex biological data, gain insights into cellular processes, and discover potential applications in fields such as drug discovery, disease research, and personalized medicine. In this study we used KEGG PATHWAY Database online server for analysis the involvement of the cyclin dependent kinase 6 gene in different cellular pathway (Chen et al., 2017).

### 2.8 *In silico* analysis

#### 2.8.1 Ligand preparation

The phytochemicals from *V. diversifolium* were retrieved in sdf format from the Pub-Chem database (Kim et al., 2021). These compounds underwent a purification process and were subjected to energy minimization using the Avogadro software, employing the mmf94 force field methodology (Hanwell et al., 2012).

#### 2.8.2 Protein preparation

The 3D structure of target proteins, namely, cyclin-dependent kinase 6 (CDK6, PDB ID: 1xo2), tyrosinase (PDB ID: 3awu), alpha-amylase (PDB ID: 3baj), alpha-glucosidase (PDB ID: 3w37), AChE (PDB ID: 4bdt), and BChE (PDB ID: 6qab) were retrieved from protein data bank (Bouley et al., 2015)**.** The protein structure was initially cleaned and heteroatoms were removed by using Discovery Studio software (Versions: 21.1.0.0). Then, using the GROMOS96 43b1 force field and the SwissPDB Viewer software, the energy of the cleaned protein was minimized and optimized (Guex and Peitsch, 1997).

#### 2.8.3 Molecular docking

Utilizing the PyRx software and its Autodock Wizard feature, a molecular docking study was performed between phytochemicals from *V. diversifolium* and the target proteins (Dallakyan and Olson, 2015). Firstly, the protein’s structural configuration was converted into a macromolecular representation, and the ligands were transformed into PDBQT format. For the subsequent docking process, specific parameters were established: the center coordinates and a grid box size defined were set on the binding site shown in Table 1. The final docking calculations were executed using PyRx, with the selection of top-ranking molecules based on their lower binding energy values. To explore the resultant binding interactions and poses, Discovery Studio software was employed.

**TABLE 1:** The centre and grid box parameter of five target proteins.

Target protein	Centre (Å)			Dimension (Å)		
	X	Y	Z	X	Y	Z
1xo2	2.9872	48.5051	136.1195	48.3649	61.2189	47.8930
3awu	−11.547	−13.495	12.2716	51.0818	53.7692	44.8066
3baj	8.3262	28.6295	50.4178	58.65.37	75.0238	59.1801
3w37	14.2008	−16.992	−28.64.65	82.9079	74.1564	79.6519
4bdt	0.4990	−40.5040	−58.7517	61.4969	87.4343	70.2044
6qab	31.8498	16.58.21	89.5222	56.6963	58.9158	74.5426

#### 2.8.4 Statistical analysis

Experiments were performed in triplicate, and differences between the extracts were compared using one way ANOVA and Tukey’s test. Graph Pad Prism (version 9.2) was used for the analysis. A *p*-value of <0.05 was considered significant.

## 3 Results and discussion

### 3.1 Bioactive constituents

The total phenolic (TPC) and flavonoids (TFC) contents of *V. diversifolium* aerial parts extracts were determined and results are presented in Table 2. The TPC of extracts was in the range of 14.09–45.69 mg GAE/g and it was in the following decreasing order; EtOH > EtOAc >70% EtOH > H_2_OH > DCM > Hexane. The TFC of extracts was in the range of 1.01–21.57 mg RE/g and it was in the following decreasing order; EtOH >70% EtOH > H_2_OH > EtOAc > DCM > Hexane. Hence, polar solvents recovered higher amount of TPC and TFC than the non-polar solvents. It was also observed that EtOH extract recovered higher TPC and TFC than hydroethanol and water extracts. This may be attributed to the presence of more nonphenolic compounds like carbohydrates and terpenes in aqueous than in other extracts. It may also be due to the possible recovery of some phenolic compounds in EtOH which may possess more phenol groups or have higher molecular weights than the phenolics in the aqueous or hydroalcoholic extracts (Do et al., 2014). In fact, recovery of phenolics from plants materials is affected by the type of solvent extraction and extraction process (Alara et al., 2021). Thus, based on results of TPC and TFC, EtOH was the most efficient extracting solvent. Furthermore, a quantitative analysis of 32 bioactive compounds of the different extracts of *V. diversifolium* was performed by HPLC-MS/MS. Results are depicted in Table 3.

**TABLE 2:** Total phenolic and flavonoid content of extracts of *Verbascum diversifolium* aerial parts.

Extracts	Total phenolic content (mg GAE/g)	Total flavonoids content (mg RE/g)
n-Hexane	14.09 ± 0.30^e^	1.01 ± 0.28^d^
Dichloromethane	26.39 ± 0.80^d^	1.38 ± 0.46^d^
Ethyl acetate	35.31 ± 2.16^b^	3.86 ± 0.49^c^
Ethanol	45.69 ± 0.70^a^	21.57 ± 0.09^a^
70% Ethanol	32.28 ± 0.24^c^	15.64 ± 0.04^b^
Water	27.28 ± 0.50^d^	4.04 ± 0.11^c^

^*^Values are reported as mean ± SD, of three parallel measurements. GAE, Gallic acid equivalents; RE, Rutin equivalents; Different letters in the same column indicate significant differences in the extracts (*p*<0.05).

**TABLE 3:** Content (µg g^−1^ of dried extract) of bioactive compounds in *Verbascum diversifolium* aerial parts extracts.

No.	Compound	Hexane	DMC	EtOAc	EtOH	70% EtOH	Water
1	Gallic acid	n.d	n.d	5.72	19.93	13.32	9.29
2	Neochlorogenic acid	n.d	2.38	6.60	49.93	31.52	27.46
3	Catechin	n.d	n.d	n.d	n.d	n.d	n.d
4	Procyanidin B2	n.d	n.d	n.d	n.d	n.d	n.d
5	Chlorogenic acid	3.88	12.26	45.24	167.99	137.11	135.27
6	4-Hydroxy benzoic acid	18.61	37.09	465.93	341.99	365.02	230.72
7	Epicatechin	n.d	n.d	n.d	n.d	n.d	n.d
8	3-Hydroxybenzoic acid	n.d	n.d	n.d	n.d	n.d	n.d
9	Caffeic acid	n.d	n.d	26,64	109.06	122.43	130.13
10	Vanillic acid	n.d	395.53	379.50	664.92	678.82	466.09
11	Syringic acid	4.68	214.30	105.39	239.59	206.57	135.61
12	Procyanidin A2	n.d	n.d	8.88	8.31	12.99	n.d
13	P-Coumaric acid	0.90	28.68	727.89	3044.01	935.17	561.84
14	Ferulic acid	1.10	26.95	27.83	18.34	17.89	12.15
15	3,5-Dicaffeoylquinic acid	n.d	0.83	1.50	5.17	3.56	1.06
16	Rutin	10.97	14.03	574.94	4280.20	1496.52	320.32
17	Isoquercitrin	2.64	7.79	718.16	1893.99	616.71	147.79
18	Delphindin 3,5 diglucoside	1.59	5.84	698.43	1686.06	567.73	139.02
19	Phloridzin	n.d	n.d	0.33	0.56	0.62	0.25
20	Quercitrin	n.d	0.42	3.38	4.46	2.96	0.42
21	Myricetin	n.d	n.d	n.d	n.d	n.d	n.d
22	Naringin	n.d	n.d	n.d	10.76	10.76	3.59
23	Kaempferol-3-glucoside	n.d	14.88	30.67	90.00	1537.38	329.92
24	Ellagic acid	n.d	n.d	n.d	10.10	9.33	n.d
25	Quercetin	0.46	0.37	6.08	20.99	20.35	2.95
26	Phloretin	n.d	n.d	n.d	n.d	n.d	n.d
27	Isorhamnetin	n.d	n.d	n.d	n.d	n.d	n.d
28	Delphindin3-galactoside	n.d	n.d	n.d	n.d	n.d	n.d
29	Cyanidin-3-glucoside	n.d	n.d	n.d	n.d	n.d	n.d
30	Petunidin-3-glucoside	n.d	n.d	n.d	n.d	n.d	n.d
31	Pelargonidin-3-rutinoside	n.d	n.d	n.d	n.d	n.d	n.d
32	Pelargonidin-3-glucoside	n.d	n.d	n.d	n.d	n.d	n.d
33	Malvidin-3-galactoside	n.d	n.d	n.d	n.d	n.d	n.d
34	Hyperoside	0.97	3.42	480.10	1154.81	468.90	120.21
35	Hesperidin	2.06	5.92	4.20	n.d	n.d	3.70
36	Kaempferol	n.d	n.d	n.d	n.d	n.d	n.d
37	Trans-cinnamic acid	6.15	71.37	35.27	26.23	19.37	16.88
Total content		54.11	842.07	842.07	13847.40	7275.02	2794.65

The highest number of compounds was obtained from the polar extracts and the least number in the hexane and DCM extracts in agreement with the pattern obtained for TPC and TFC. The concentration of total identified compounds was in the following decreasing order; EtOH (13847.40 μg g^−1^) > 70% EtOH (7275.02 μg g^−1^) > EtOAc (4352.67 μg g^−1^) > H_2_OH (2794.65 μg g^−1^) > DCM (842.07 μg g^−1^) > Hexane (54.11 μg g^−1^). The EtOH extract predominated by the presence of rutin (4280.20 μg g^−1^), *p*-coumaric acid (3044.01 μg g^−1^), isoquercitrin (1893.99 μg g^−1^), delphindin 3,5 diglucoside (1686.06 μg g^−1^) and hyperoside (1154.81 μg g^−1^). The 70% EtOH extract was characterized by high accumulation of kaempferol-3-glucoside (1537.38 μg g^−1^), rutin (1496.52 μg g^−1^) and *p*-coumaric acid (935.17 μg g^−1^). The highest content of 4-hydroxy benzoic acid (465.93 μg g^−1^) and ferulic acid (27.83 μg g^−1^) were identified in the EtOAc extract. Highest accumulation of caffeic acid (130.13 μg g^−1^) and trans-cinnamic acid (71.37 μg g^−1^) was observed in the aqueous and DCM extracts respectively. However, most of these compounds which are known for their beneficial effect are previously identified in other *Verbascum* species (El Gizawy et al., 2019; Angeloni et al., 2021; Gökmen et al., 2021; Zengin et al., 2021; Zengin et al., 2023). Besides, bioactives like rutin, isoquercitrin and kaempferol are known to exhibit antioxidant potential while phenolic acids like vanillic, caffeic, cinnamic, 4-Hydroxy benzoic and gallic acids are effective anticancer agents and enzyme inhibitors (Abotaleb et al., 2020; Aleixandre et al., 2022; Budryn et al., 2022).

### 3.2. Chemical antioxidant assays

Six complementary assays including radical scavenging (DPPH and ABTS), reducing (CUPRAC and FRAP), metal chelating and total antioxidant assays were performed to evaluate the antioxidant property of *V. diversifolium* aerial parts and results are presented in Table 4. Extracts showed similar pattern in their scavenging towards the DPPH and ABTS radicals but recorded higher values in ABTS assay. The DPPH radical scavenging of different extracts was in the following decreasing order; EtOH (46.86 mg TE/g) ≂ 70% EtOH (46.33 mg TE/g) (*p* ≥ 0.05) > EtOAc (38.49 mg TE/g) > H_2_OH (37.25 mg TE/g) > DCM (1.67 mg TE/g) > Hexane (0.54 mg TE/g) (*p* < 0.05). The EtOH, 70% EtOH and aqueous extracts had relatively the same effect to scavenge the ABTS radical (73.56–73.03 mg TE/g) (*p* ≥ 0.05) followed respectively by the EtOAc (53.33 mg TE/g), DCM (22.56 mg TE/g) and Hexane (8.10 mg TE/g) extracts (*p* < 0.05). The highest result to reduce the Cu^++^ and Fe^+++^ ions was exerted respectively by the EtOH (174.84 and 79.24 mg TE/g respectively), EtOAc (128.66 and 60.83 mg TE/g respectively) and 70% EtOH (116.08 and 57.47 mg TE/g) extracts (*p* < 0.05). For the DCM extract, it significantly (*p* < 0.05) displayed higher Cu^++^ ions reducing effect (89.85 mg TE/g) than the aqueous extract (77.52 mg TE/g) while the opposite was true for their effect to reduce the Fe^+++^ ions (30.22 and 51.40 mg TE/g respectively). The hexane extract showed the least values in both assays. Although the EtOH extract exhibited the highest antioxidant result in the 4 previous assays, it nonetheless displayed the least metal chelating power (13.79 mg EDTAE/g). The aqueous extract (54.63 mg EDTAE/g) exerted significantly (*p* < 0.05) the highest value followed by the DCM (42.92 mg EDTAE/g) extract. The 70% EtOH (35.67 mg EDTAE/g) and hexane (35.12 mg EDTAE/g) extracts showed relatively the same effect (*p* ≥ 0.05).

**TABLE 4:** Antioxidant properties of *Verbascum diversifolium* aerial parts extracts.

Extracts	DPPH (mg TE/g)	ABTS (mg TE/g)	CUPRAC (mg TE/g)	FRAP (mg TE/g)	PBD (mmol TE/g)	MCA (mg EDTAE/g)
n-Hexane	0.54 ± 0.04^e^	8.10 ± 0.93^d^	38.82 ± 1.93^f^	13.85 ± 0.56^f^	0.97 ± 0.07^e^	35.12 ± 2.55^c^
Dichloromethane	1.67 ± 0.15^d^	22.56 ± 1.95^c^	89.85 ± 0.17^d^	30.22 ± 0.97^e^	2.08 ± 0.07^c^	42.92 ± 1.34^b^
Ethyl acetate	38.49 ± 0.67^b^	53,.3 ± 1.89^b^	128.66 ± 3.45^b^	60.83 ± 0.78^b^	2.58 ± 0.12^a^	23.93 ± 0.55^d^
Ethanol	46.86 ± 0.08^a^	73.56 ± 0.49^a^	174.84 ± 6.70^a^	79.24 ± 0.62^a^	2.37 ± 0.01^b^	13.79 ± 2.35^e^
70% Ethanol	46.33 ± 0.18^a^	73.42 ± 0.12^a^	116.08 ± 0.28^c^	57.47 ± 1.55^c^	1.55 ± 0.05^d^	35.67 ± 2.19^c^
Water	37.25 ± 1.68^c^	73.03 ± 0.01^a^	77.52 ± 1.68^e^	51.40 ± 0.13^d^	1.47 ± 0.07^d^	54.63 ± 0.44^a^

^*^Values are reported as mean ± SD, of three parallel measurements. PBD, phosphomolybdenum; MCA, metal chelating activity; TE, trolox equivalent; EDTAE, EDTA, equivalent; na, not active.

Chelation of metal ions is considered a good strategy to avoid ROS generation that is associated with redox active metal catalysis. The transition metal ion, Fe^2+^, has the ability to move single electrons, stimulate lipid peroxidation by the Fenton reaction and thus can allow for the formation and propagation of many radical reactions (Kumar et al., 2013a; Kumar et al., 2013b). Ferrozine can quantitatively form complexes with Fe^2+^; however, in the presence of a chelating milieu, the formation of Fe^2+^-ferrozine complex is decreased, suggesting chelation of iron by phytochemicals present in the plant (Sunday Joel et al., 2017). Phytochemicals present in *V. diversifolium* extracts interfered with the formation of ferrous-ferrozine complex, suggesting that they had chelating activity and captured ferrous ion before ferrozine (Mishra et al., 2013). It was postulated that the reason that extracts showed higher reducing values for copper ions than for iron ions can be explained in terms of the standard reduction potentials of the metals. The standard reduction potential of the Cu^2+^/Cu^+^ couple (+0.15 V) is much lower than that for the Fe^3+^/Fe^2+^ couple (+0.77 V) (Lomozová et al., 2022).

The total antioxidant results of extracts was in the range of 0.97 and 2.58 mmol TE/g with least and highest significant (*p* < 0.05) activity recorded from the hexane and EtOAc extracts respectively. It is clear that extracts showing high antioxidant results are rich in TPC supporting previous studies that correlating the antioxidant activity of extracts to their phenolic content (Alara et al., 2021; Molole et al., 2022). Furthermore, phenolic compounds like chlorogenic, 4-hydroxy benzoic, vanillic, syringic, *p*-coumaric, caffeic, and cinnamic acids, rutin, isoquercitrin, kaempferol-3-glucoside and hyperoside, detected in considerable amount in polar extracts, are well known for their antioxidant properties (Brewer, 2011; Rababah et al., 2011; Spiegel et al., 2020). Furthermore, the antioxidant effect of phenolics is highly associated with their structure, as structures with the most hydroxyl groups usually exert the greatest antioxidant activity (Rice-Evans et al., 1996). It is known that hydroxylated cinnamates are more effective than benzoate counterparts. Insertion of an ethylenic group between a phenyl ring carrying a *p*-hydroxyl group and the carboxylate group, as in *p*-coumaric acid, has a highly favourable effect on the reducing properties of the OH group compared with cinnamic acid. Incorporation of a hydroxyl group into *p*-coumaric acid adjacent to that in the para position as in caffeic acid increase its antioxidant property. Substitution of the 3-hydroxyl group of caffeic acid by a methoxy group (ferulic acid) considerably enhances its antioxidant effectiveness (Rice-Evans et al., 1996). Also, it has been established that the position and degree of hydroxylation is fundamental to the antioxidant activity of flavonoids, particularly in terms of the *o*-dihydroxylation of the B ring, the carbonyl at position 4, and a free hydroxyl group at positions 3 and/or 5 in the C and A tings, respectively (Rice-Evans et al., 1996). Eventually, these compounds may act synergistically and the highest antioxidant effect displayed by polar extracts, mainly the EtOH extract, could be attributed to relatively high accumulation of these compounds.

Overall, it was noted that phytoconstituents with highest antiradical and ion reducing results were mainly recovered from the EtOH extract, while those with best chelating and total antioxidant results were mainly accumulated in the aqueous and EtOAc extracts, respectively. This indicates that *V. diversifolium* contain antioxidant molecules with variable mechanisms of action. However, these results supported those obtained for *V. nubicum* (El Gizawy et al., 2019), *V. bugulifolium* (Gökmen et al., 2021), *V. bombyciferu* (Angeloni et al., 2021), *V. euphraticum, V. oocarpum*, *V. cheiranthifolium, V. myriocarpum* and *V. pyroliforme* (Zengin et al., 2021; Zengin et al., 2023) where the highest antioxidant activity was mainly exerted by polar extracts (MeOH, hydromethanolic or aqueous). Also, the two compounds, verbascoside and luteolin, isolated from the aerial parts of *V. bugulifolium* were found to possess potent antioxidant activity in the DPPH (IC_50_ = 5 μg/mL for luteolin) and CUPRAC (IC_50_ = 10 µg/mLfor verbascoside) assays (Gökmen et al., 2021). Hence this genus can be considered as a promising source of antioxidants.

### 3.3 Enzyme inhibition activity

Enzymes are currently receiving increased attention due to their potential therapeutic alternative for several diseases like Alzheimer’s disease, diabetes and some skin disorders. In the present study, extracts of *V. diversifolium* aerial parts were evaluated for their capacity to inhibit the AChE, BChE, Tyrosinase, α-amylase and α-glucosidase enzymes. Results are shown in Table 5.

**TABLE 5:** Enzyme inhibitory effects of *Verbascum diversifolium* aerial parts extracts.

Extracts	AChE (mg GALAE/g)	BChe (mg GALAE/g)	Tyrosinase (mg KAE/g)	Amylase (mmol ACAE/g)	Glucosidase (mmol ACAE/g)
n-Hexane	2.38 ± 0.16^b^	2.54 ± 0.10^d^	41.40 ± 2.58^d^	0.35 ± 0.01^c^	1.00 ± 0.01^c^
Dichloromethane	2.28 ± 0.02^b^	5.21 ± 0.01^a^	54.54 ± 2.23^ab^	0.51 ± 0.01^a^	0.96 ± 0.02^c^
Ethyl acetate	1.12 ± 0.34^d^	3.09 ± 0.24^c^	46.74 ± 4.08^cd^	0.41 ± 0.01^b^	1.01 ± 0.05^bc^
Ethanol	2.66 ± 0.01^a^	4.64 ± 0.29^b^	58.39 ± 1.32^a^	0.25 ± 0.01^d^	1.07 ± 0.01^ab^
70% Ethanol	2.64 ± 0.02^a^	2.63 ± 0.13^d^	50.93 ± 1.51^bc^	0.23 ± 0.01^d^	1.09 ± 0.01^a^
Water	1.46 ± 0.06^c^	na	na	0.04 ± 0.01^e^	na

^**^Values are reported as mean ± SD, of three parallel measurements. GALAE, Galantamine equivalent; KAE, Kojic acid equivalent; ACAE, Acarbose equivalent; na, not active.

The AChE inhibition activity of different extracts was in the range of 1.12–2.66 mg GALAE/g with highest activity displayed by the EtOH and 70% EtOH extracts (*p* > 0.05) followed by the hexane (2.38 mg GALAE/g) and DCM (2.28 mg GALAE/g) extracts (*p* > 0.05). The aqueous and EtOAc extracts revealed the least activity. With the exception of the aqueous extract, all extracts exerted good anti-BChE activity in the range of 2.54–5.21 mg GALAE/g. The highest significant (*p* < 0.05) effect was displayed by the DCM extract followed respectively by the EtOH (4.64 mg GALAE/g) and EtOAc (3.09 mg GALAE/g) extracts. It was noted that most extracts showed higher anti-BChE activity than anti-AChE one.

Some of the identified phenolics in the present study were shown to possess significant enzyme inhibition property. For example, the anti-AChE activity of phenolic acids was reported in the following order; rosmarinic acid > caffeic acid > gallic acid = chlorogenic acid > homovanillic acid > sinapic acid (Szwajgier, 2015). Additionally, syringic, vanillic, 4-Hydroxy benzoic and gallic acids were reported to exhibit anti-AChE activity (Srinivasulu et al., 2018; Budryn et al., 2022). The flavonoids, quercetin and kaempferol were also reported as efficient AChE inhibitors (Szwajgier, 2015). All extracts, except the aqueous one, exhibited considerable anti-Tyrosinase activity in the range of 41.40–58.39 mg KAE/g with the EtOH and hexane extracts revealed the highest and least activity. This activity could be partly due the presence of *p*-coumaric acid and caffeic acid (Crespo et al., 2019). Although these compounds were also detected in the aqueous extract it devoid of anti-Tyrosinase activity suggesting an antagonistic effect by other molecules. Targeting the α-amylase and α-glucosidase enzymes is believed an important approach in the development of novel anti-diabetic agents where by postprandial hyperglycemia can be better controlled (Alam et al., 2019). The α-amylase inhibition activity was in the range of 0.04–0.51 mmol ACAE/g with highest and least activity exerted by the DCM and aqueous extracts. The α-glucosidase inhibition activity was in the range of not active to 1.09 mmol ACAE/g where the EtOH and 70% EtOH extracts revealed the highest activity and the aqueous extract was not effective. A recent study showed that syringic and vanillic acids were effective against the α-amylase while caffeic acid was effective against the α-glucosidase (Aleixandre et al., 2022). Overall, these results revealed that *V. diversifolium* possessed significant enzyme inhibition properties against the tested enzyme in agreement with previous studies on other *Verbascum* species (Angeloni et al., 2021; Zengin et al., 2021; Kızıltaş et al., 2023; Zengin et al., 2023).

### 3.4 Cytotoxicity

**FIGURE 1:**
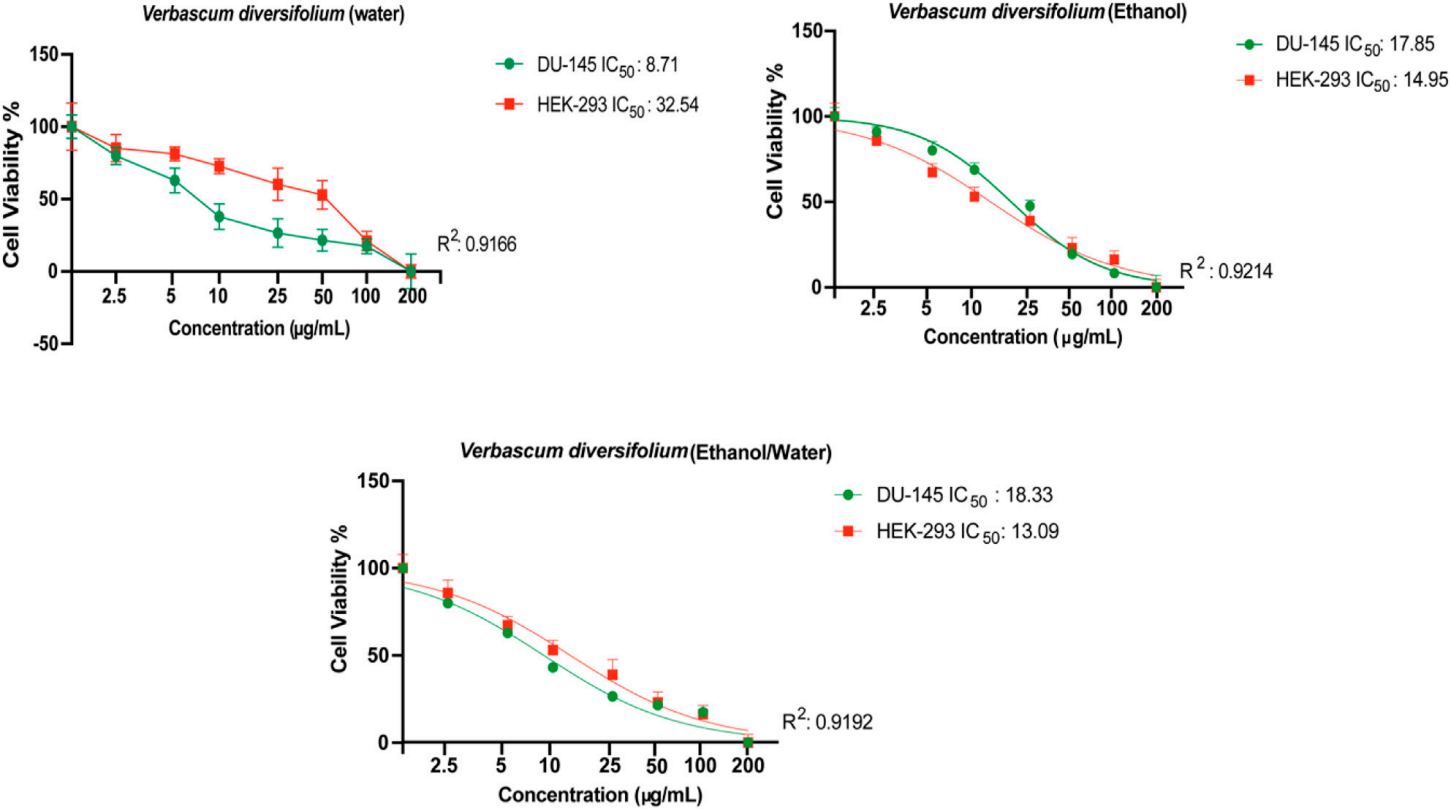
Evaluation of cytotoxic effects of *Verbascum diversifolium* (ethanol, ethanol/water and water) extracts on DU-145 and HEK-293 cell lines by MTT assay. All data are expressed as the mean ± SD from three independent experiments. The IC_50_ value was calculated using the GraphPad Prism 9 program.

The search for new natural anticancer drugs is a main objective of scientific research (El Hasasna et al., 2015; El Hasasna et al., 2016; Athamneh et al., 2017; Alsamri et al., 2019; Athamneh et al., 2020). In the present study, the cytotoxic effect of the polar (EtOH, 70% EtOH and H_2_OH) extracts of *V. diversifolium* aerial parts was examined against 5 cancer cells; DU-145, MDA-MB-231, HELA, HT-29 and HGC-27 in addition to the normal cell HEK-293. Results are presented in Table 6 and Figure 1. According to the US National Cancer Institute (NCI) plant screening program, a crude plant extract is generally considered to have acceptable *in vitro* cytotoxic activity if the IC_50_ value is less than 20 μg/mL after 48- and 72-h incubation with cancer cell lines (Boik, 2001). Based on the present results, the three extracts displayed cytotoxic effect against DU-145 cell (IC_50_ 8.71–17.85 μg/mL) with the highest effect obtained from the aqueous extract. Results also revealed cytotoxicity against HGC-27 (IC_50_ 7.051–16.09 μg/mL) with the EtOH extract exerting the highest effect. Additionally, the 70% EtOH (IC_50_ 7.65 μg/mL) and aqueous (IC_50_ 13.89 μg/mL) extracts showed cytotoxicity against HELA cells. All the three extracts were less toxic towards the MDA-MB-231 and HT-29 cells (IC_50_ > 20 μg/mL). On the other hand, the EtOH (IC_50_ 14.95 μg/mL) and 70% EtOH (IC_50_ 13.09 μg/mL) extract were toxic against the normal HEK-293 cell while the aqueous extract was significantly less toxic (IC_50_ 32.54 μg/mL). Besides, the selectivity index (SI) was calculated and SI > 3 is considered as indicative for safer and more effective therapy in cancer therapy (Segun et al., 2019). Accordingly, the aqueous extract was found to be the most selective to inhibit the DU-145 cell with SI = 3.7 while the 70% EtOH (SI = 1.7) and EtOH (SI = 2.1) extracts were less selective for HELA and HGC-27 cell. However, assessing the clinical relevance of these findings is still warranted.

**TABLE 6:** Cytotoxic effects of *Verbascum diversifolium* polar extracts on cancer and normal cell lines.

Cell lines	IC_50_ (µg/mL)		
	Ethanol	70% Ethanol	Water
DU-145	17.85	18.33	8.71
MDA-MB-231	114.2	47.84	82.59
HELA	21.94	7.652	13.89
HT-29	40.11	86.42	27.35
HGC-27	7.051	10.99	16.09
HEK-293	14.95	13.09	32.54

Phenolic compounds exert a wide variety of anticancer effects. They modulate ROS-scavenging enzyme activities, participate in arresting the cell cycle, induce apoptosis, autophagy, and suppress cancer cell proliferation and invasiveness. The anticancer activity of phenolic acids and flavonoids is highly associated with their chemical structure. For example, an increase in the number of the OH ring substituents in the phenolic acids leads to higher antiproliferative and cytotoxic activities (Abotaleb et al., 2020). For flavonoids, the cytotoxic activity is also affected by the number of hydroxyl groups that the compounds carry; and the saturation of the C2 = C3 double bond reduces the activity (Gomes et al., 2003). However, some of the compounds identified in the present study are known to exert cytotoxic effects against cancer cell lines. For instance, coumaric acid exerted cytotoxic effect against human colon HT-29 and HCT-15, human lung A549 and prostate PC-3 cells (Sharma et al., 2017; Sharma et al., 2018; Sharma et al., 2019). Vanillic acid was active against human colon cancer HCT116 cell (Gong et al., 2019). Syringic acid possesses cytotoxic effect in human colorectal cancer SW1116 and SW837 cells (Abaza et al., 2013). Gallic and caffeic acids were effective against HeLa cells (Gomes et al., 2003). The latter also exhibited cytotoxic effect towards Ht-29 cell line (Anantharaju et al., 2016). Quercetin has antitumor activity against colon carcinoma CT-26 cells, prostate adenocarcinoma LNCaP cells, PC3 cells, pheochromocytoma PC12 cells, estrogen receptor-positive breast cancer MCF-7 cells, acute lymphoblastic leukemia MOLT-4 T-cells, human myeloma U266B1 cells, human lymphoid Raji cells, and ovarian cancer CHO cells (Hashemzaei et al., 2017). Moreover, many *Verbascum* species were reported in the literature to exert significant cytotoxic effect against many cancer cell lines like *V. sinaiticum* against P-388 cells (Afifi et al., 1993), MCF-7, and HepG2 cells (Tauchen et al., 2015), *V. pterocalycinum* var. *mutense* against SK-MEL cells (Tatli and Akdemir, 2006b), *V. sinaiticum* against Hep-2 and MCF-7 cells. (Talib and Mahasneh, 2010), *V. thapsus* against A549 cells (Zhao et al., 2011)*, V. ovalifolium* against SK-MEL-2 cell (Luca et al., 2019), *V. nigrum* against A431 cells (Iliescu et al., 2020), *V. alceoides* against HeLa and HUVEC cells (Dinani et al., 2020), and *V. lasianthum* against A549 cells (Hazman et al., 2021).

### 3.5 Selection of target protein and its Kyoto Encyclopedia of genes and genomes pathway analysis

**FIGURE 2:**
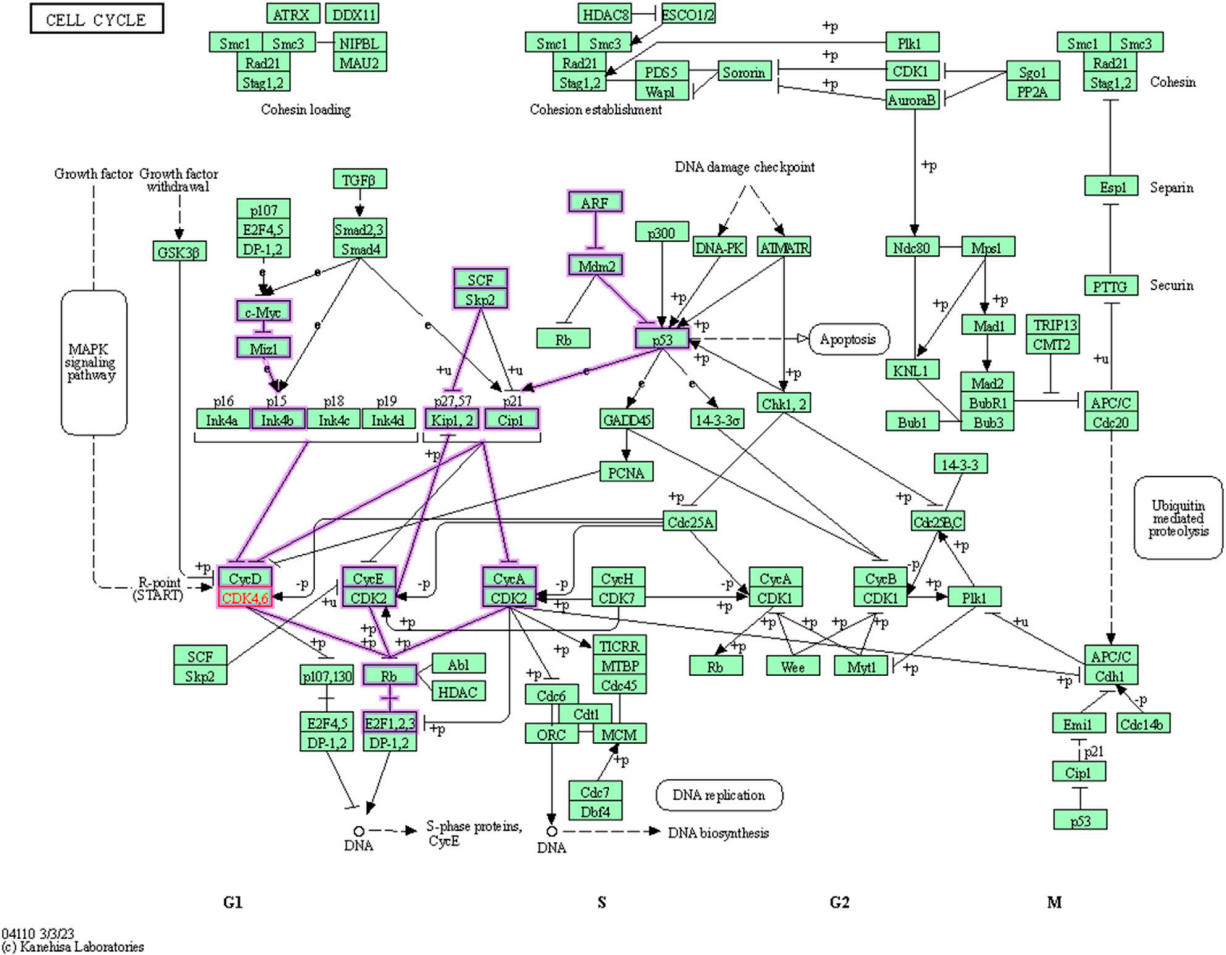
Involvement of the cyclin dependent kinase 6 in Cell cycle pathway.

Cyclin dependent kinase 6 (CDK6) assumes a central position in the regulation of the cell cycle, particularly within the G1 phase, where it engages in partnerships with cyclins to propel the progression of the cell cycle (Figure 2). Conducting a KEGG pathway analysis on CDK6 offers an opportunity to gain valuable insights into the biological mechanisms and pathways where this protein plays a crucial role. To perform the *in silico* anticancer activity, CDK6 (PDB ID: 1XO2) was selected as a potential drug target and its KEGG pathway analyzed. The KEGG identifier of this protein is hsa:1021. This target gene plays a significant role in 23 different pathways as shown in Supplementary Table S1.

**FIGURE 3:**
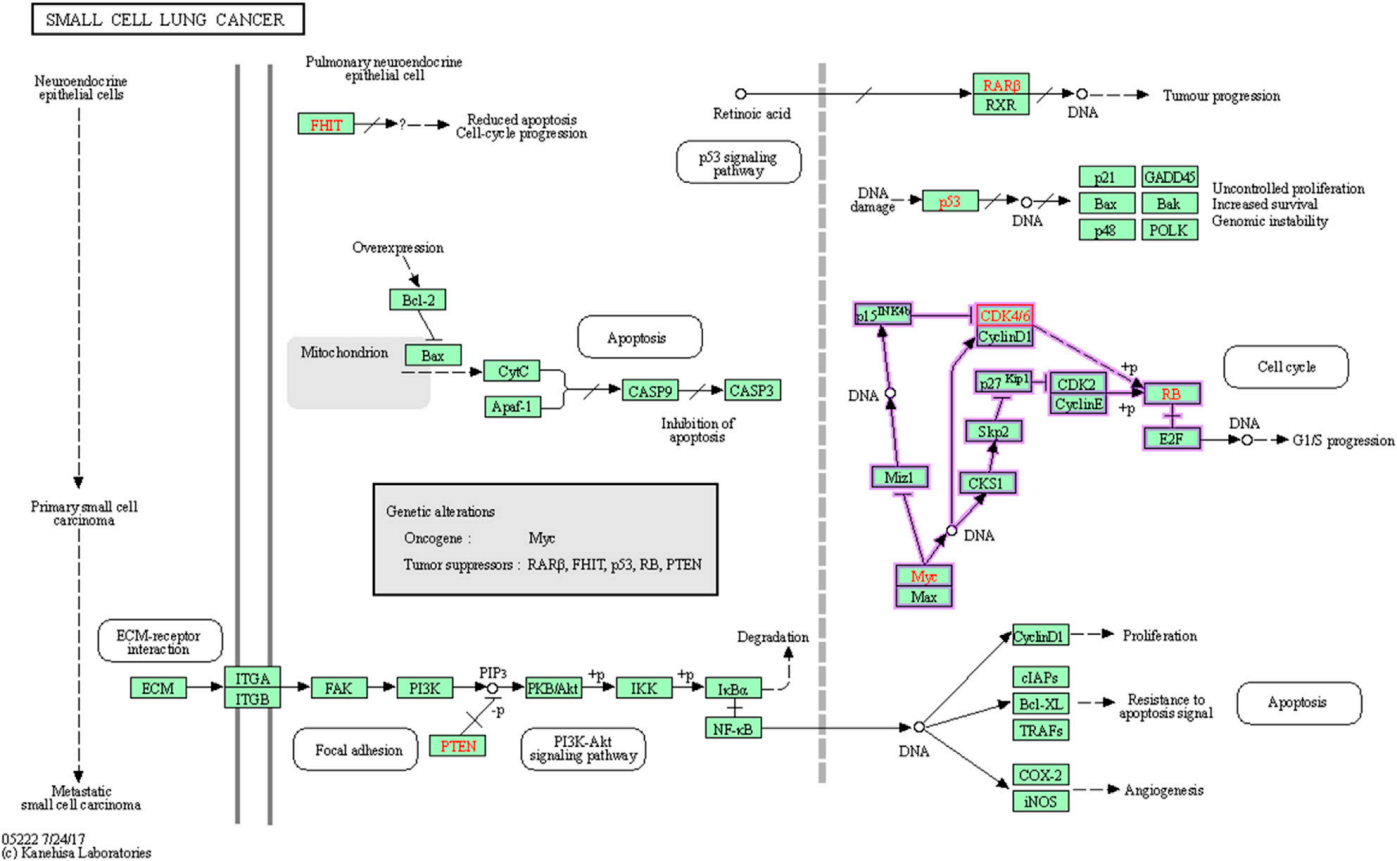
Involvement the cyclin dependent kinase 6 in Small cell lung cancer.

CDK6 holds promise as a viable target for cancer treatment, as indicated in Figure 3. Employing KEGG pathway analysis can shed light on the pathways linked to CDK6’s function, which could have significance in the realm of drug development. This analysis has the potential to assist in the discovery of new drug targets, the exploration of possible combination therapies, and the comprehension of how currently available drugs targeting CDK6 may influence other pathways.

### 3.6 Molecular docking targeting cyclin dependent kinase 6

**FIGURE 4:**
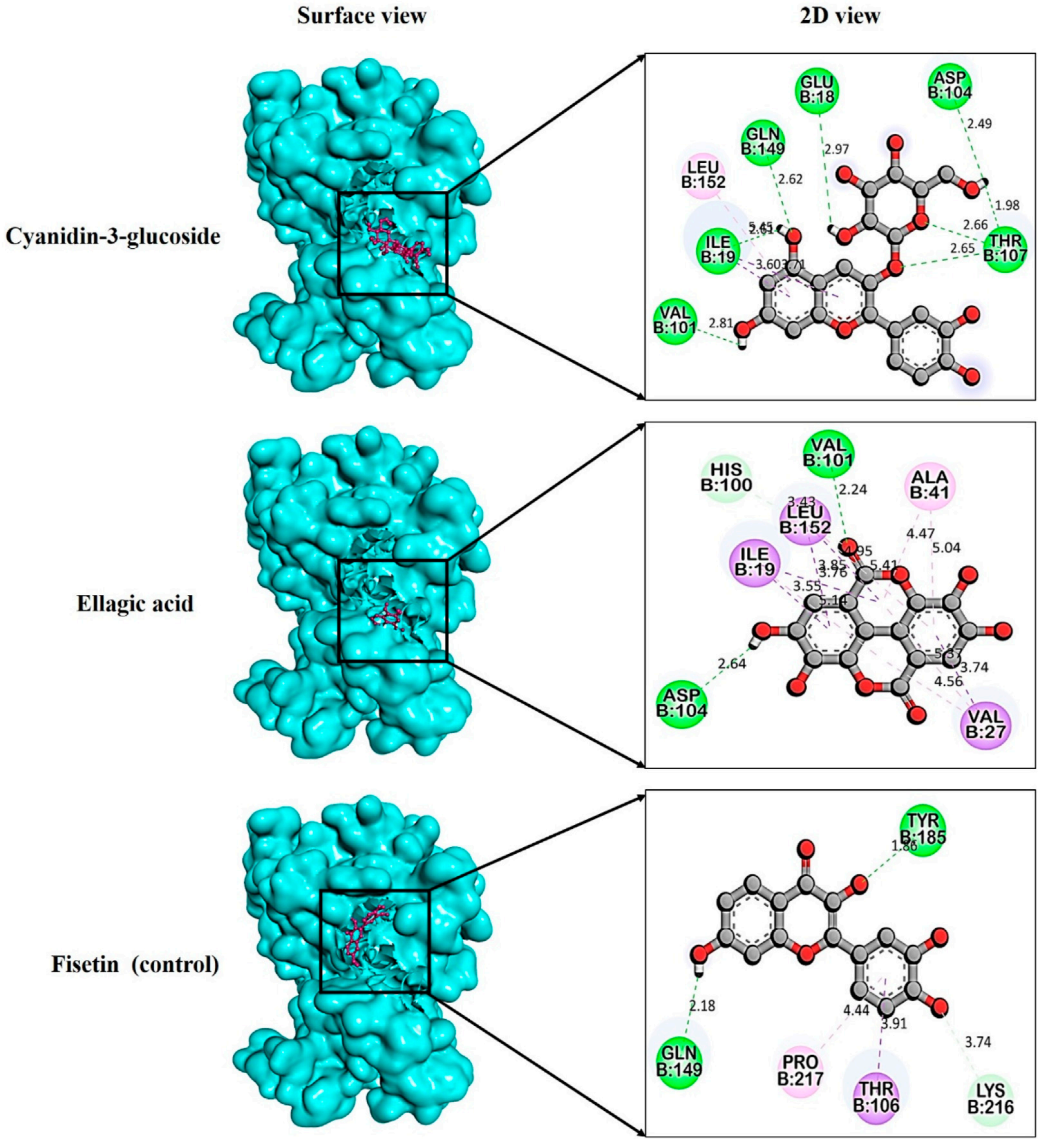
Docking pose targeting cyclin dependent kinase 6 (PDB ID: 1xo2) with phytochemicals from *Verbascum diversifolium*.

After the comprehensive KEGG pathway analysis, we confirmed that CDK6 is involved in different cancer pathways that emerge it as potential anticancer drug target. Therefore, in this study, we sought to determine if phytochemicals of *V. diversifolium* can act as potential inhibitors of this target. Our results revealed that cyanidin-3-O-glucoside showed the highest binding affinity with −10.2 kcal/mol compared to the control compounds. Fisetin with −8.87 kcal/mol, where it interacts the target protein 1xo2 with six hydrogen bonds at Glu18, Ile19, Val101, Asp104, Thr107, and Gln149 (Figure 4A). Similarly, the complex between 1xo2 and ellagic acid exhibited two hydrogen bond exactly the same position as cyanidin-3-O-glucoside at Val101 and Asp104 (Figure 4B) with binding energy −9.1 kcal/mol (Supplementary Table S2).

### 3.7 Molecular docking targeting tyrosinase

Tyrosinase and its associated proteins play a key role in melanin production, and inhibiting tyrosinase is a well-established approach for addressing hyperpigmentation issues. Many natural and synthetic antimelanogenic agents have shown promise in research, but often present with side effects like depigmentation, dermatitis, thyroid cancer, nephrotoxicity and genotoxicity (Maeda and Fukuda, 1996; Westerhof and Kooyers, 2005; Parvez et al., 2006; Chen et al., 2015; Ogiwara et al., 2015). Consequently, identifying new melanogenesis inhibitors is very important in medicine and cosmetics.

**FIGURE 5:**
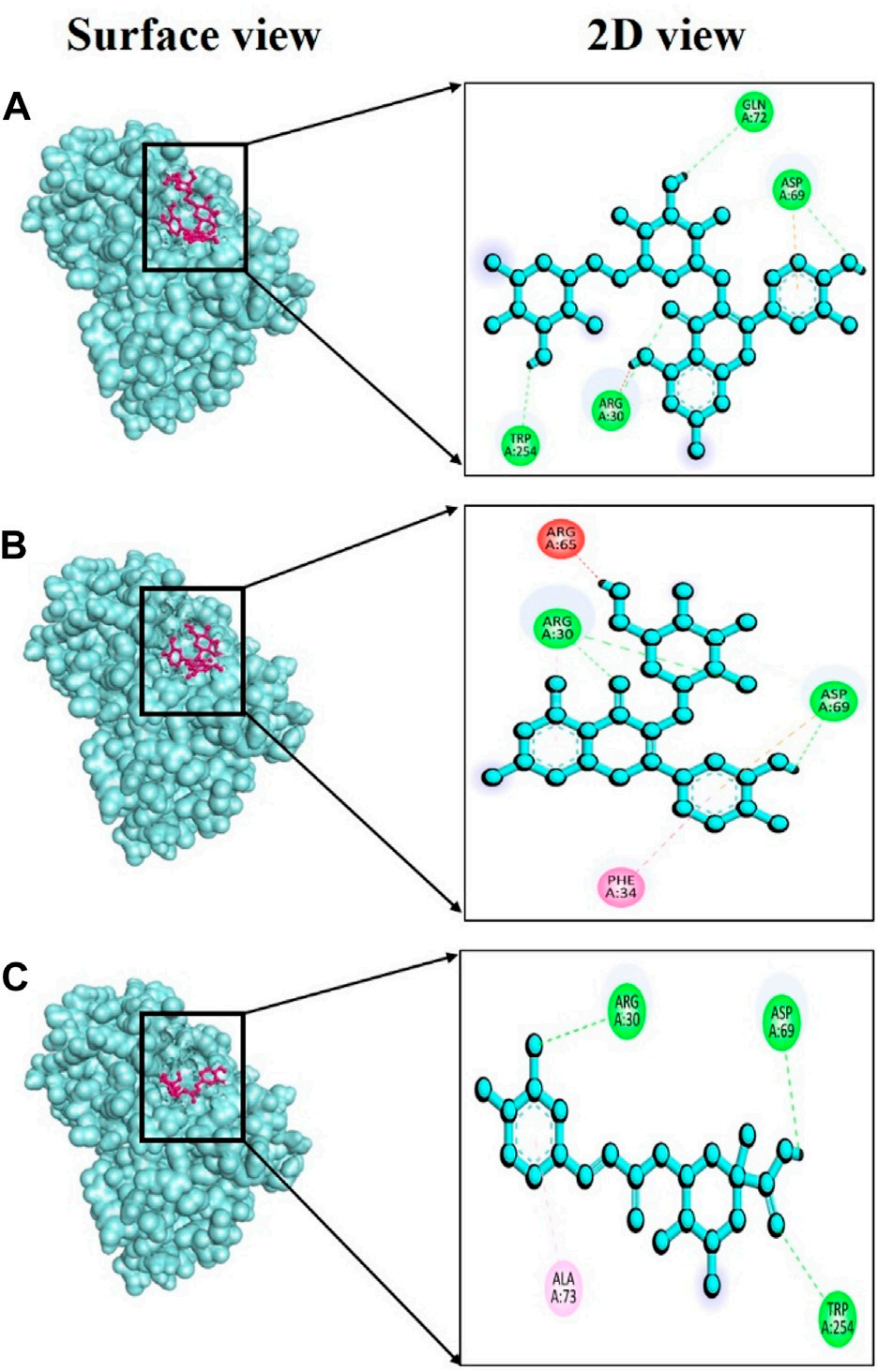
Docking interaction targeting tyrosinase (PDB ID: 3awu), where **(A)** Isoquercetin, **(B)** Rutin, **(C)** Neochlorogenic acid.

In this study, specific phytochemicals derived from *V. diversifolium* were docked as ligands targeting tyrosinase (PDB ID: 3awu) for assessing the inhibitory activity. Among the three complexes, 3awu + Isoquercetin exhibited the strongest binding affinity of −7.6 kcal/mol followed by 3awu + Rutin and 3awu + Neochlorogenic acid with binding affinity of −7.5 and −7.3 kcal/mol (Supplementary Table S3), respectively. Moreover, a total of four strong hydrogen bonds were identified for complex 3awu + Isoquercetin at Arg30, Asp69, Gln72, and Trp254 residues with bond length of approximately 1.25, 3.04, 2.84, and 2.59 Å (Figure 5A), correspondingly. The complex 3awu + Rutin revealed two hydrogen bonds at Arg30 (2.74Å) and Asp69 (2.57Å) residues (Figure 5B), respectively. Lastly, with a slightly lower binding affinity than other complexes, 3awu + Neochlorogenic acid displayed three hydrogen bonds at Arg30 (2.63Å), Asp69 (2.46Å) and Trp254 (2.86Å) residues (Figure 5C), in that order. The numerous hydrogen bonds identified in this molecular docking study provide valuable insights into the binding interactions between the ligand and the target protein, supporting the potential therapeutic relevance of the studied compounds.

### 3.8 Molecular docking targeting alpha-amylase

**FIGURE 6:**
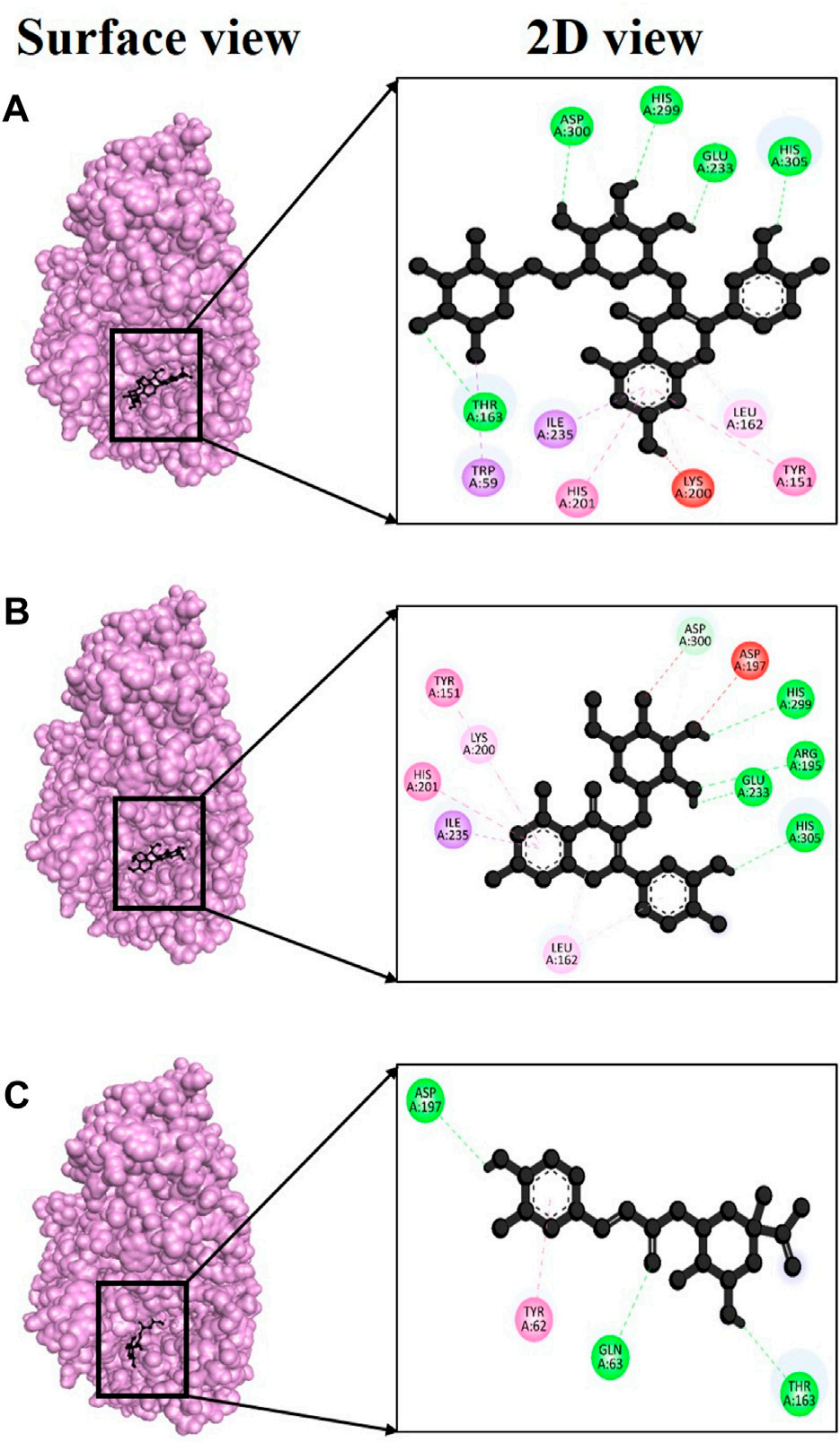
Docking interaction targeting alpha-amylase (PDB ID: 3baj), where **(A)** Isoquercetin, **(B)** Rutin, **(C)** Neochlorogenic acid.

Over time, various phenolic compounds have gained recognition for their strong antioxidant properties and their potential in managing diabetes mellitus. Examples of these plant phenolics include esculetin, umbelliferone, bergapten, emodin, and kaempferol, all of which have previously been studied for their ability to regulate hyperglycemia (Lin et al., 2016). Despite their promising potential, there have been fewer studies on the effects of these compounds from *V. diversifolium*. Therefore, in this study, we conducted *in silico* molecular docking assays to assess the alpha-amylase (PDB ID: 3baj) inhibitory activities of compounds isolated from *V. diversifolium*. Among the three complexes, 3baj + Rutin showed exceptionally strong binding energy of −9.4 kcal/mol (Supplementary Table S4) which indicates a robust interaction between the two molecules, and suggesting a potentially promising ligand-protein complex. Furthermore, this complex presented five hydrogen bonds at Thr163, Glu233, His299, Asp300 and His305 amino acid residues with bonding distance of 2.55, 2.83, 2.34, 2.19, and 2.13 Å (Figure 6A), correspondingly. The complex 3baj + Isoquercetin showed a binding affinity of −8.7 kcal/mol (Supplementary Table S4 **)** revealing four hydrogen bonds at Arg195 (2.85 Å), Glu233 (2.67Å), His299 (2.60Å) and His305 (2.76Å) residues (Figure 6B) which also signifies a significant and favorable interaction between the two molecules. Lastly, 3baj + Neochlorogenic acid complex gave −7.9 kcal/mol binding affinity. Within this complex, three hydrogen bonds were identified at Gln63 (2.59Å), Thr163 (2.60Å), and Asp187 (2.56Å) residues (Figure 6C), respectively.

### 3.9 Molecular docking targeting Alpha-glucosidase

**FIGURE 7:**
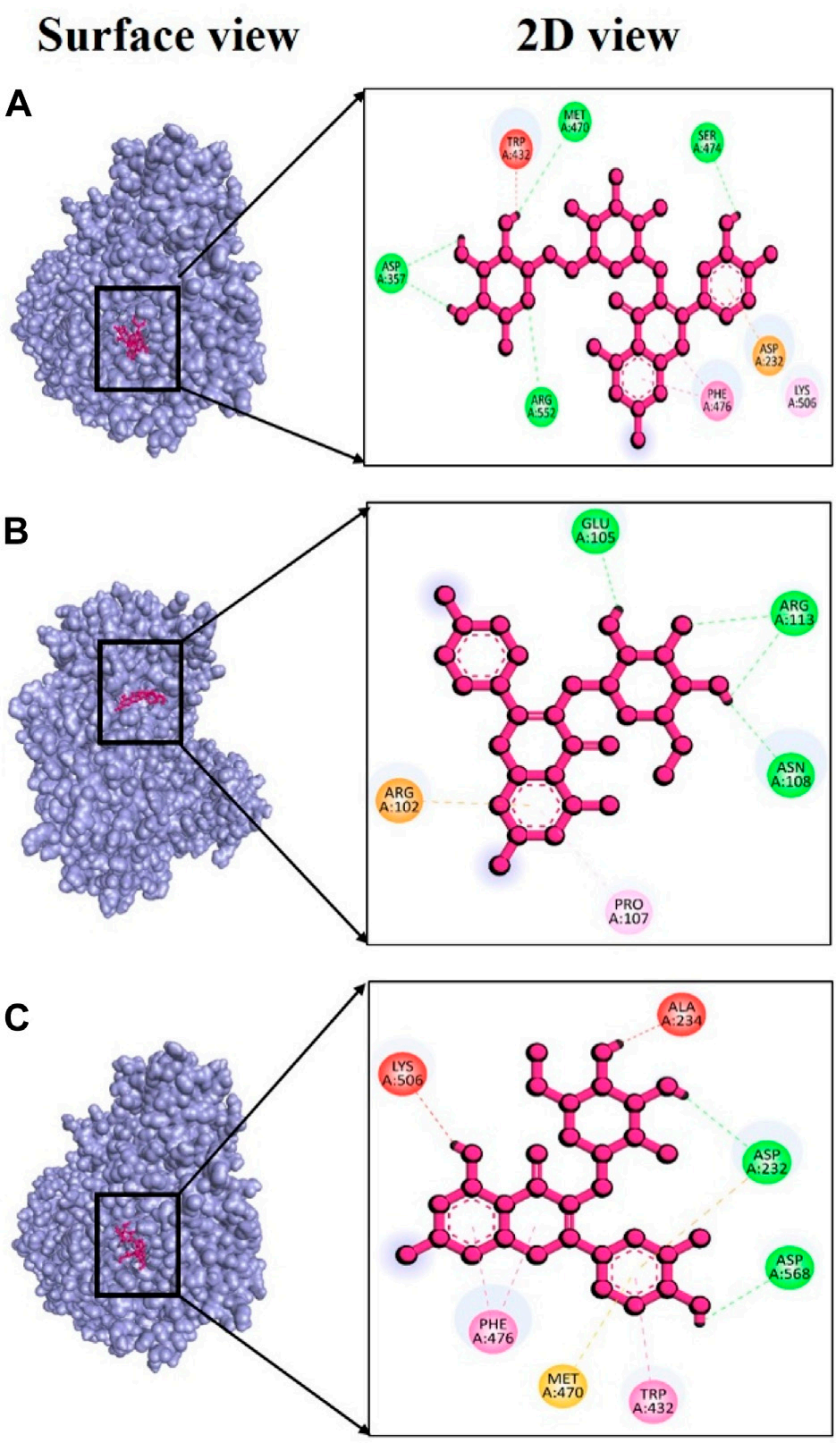
Docking interaction targeting alpha-glucosidase (PDB ID: 3w37), where **(A)** Rutin, **(B)** kaempferol-3-glucoside, **(C)** Isoquercetin.

Alpha-glucosidase (PDB ID: 3w37) is another critical enzyme involved in diabetes mellitus. Compounds capable of inhibiting alpha-glucosidase can slow down the digestion and absorption of carbohydrates. Consequently, they can help regulate postprandial hyperglycemia independently of insulin (Kazeem et al., 2013). Several types of synthetic medications like metformin, sulfonylureas, thiazolidinediones, biguanides, meglitinides, and dipeptidyl peptidase-IV inhibitors have already been developed to treat type 2 diabetes mellitus (Stein et al., 2013; Quan et al., 2019). However, prolonged use of these therapeutic agents may lead to severe side effects, including liver complications, hypoglycemia, and diarrhea, among others. Given these concerns, it has become essential to develop new inhibitors with high effectiveness and minimal toxicity. In this context, we assessed, using *in silico* docking approaches, whether phytochemicals from *V. diversifolium* possess inhibitory properties of Alpha-glucosidase. Among the three complexes studied, the interaction between 3w37 and rutin exhibited an exceptionally strong binding energy of −9.5 kcal/mol (Supplementary Table S5) indicating a dynamic interaction between the two molecules. This suggests a potentially promising ligand-protein complex. Additionally, in this complex, four hydrogen bonds were observed at Asp357, Met470, Ser474, and Arg552 amino acid residues, with bonding distances of 2.11, 2.94, 2.36, and 3.05 Å (Figure 7A), respectively. The complex involving 3w37 and kaempferol-3-glucoside showed a binding affinity of −7.4 kcal/mol (Supplementary Table S5), demonstrating a favorable interaction between the two molecules. This complex featured three hydrogen bonds at Glu105 (2.19 Å), Asn108 (2.30 Å), and Arg113 (2.55 Å) residues (Figure 7B), further emphasizing the significance of their interaction. Finally, the complex 3w37+ Isoquercetin had a binding affinity of −7.2 kcal/mol (Supplementary Table S5). Within this complex, two hydrogen bonds were identified at Asp232 (2.52 Å) and Asp568 (1.93 Å) residues (Figure 7C), contributing to the overall stability of the complex.

### 3.10 Molecular docking targeting AChE and BChE

Alzheimer’s disease (AD) is a progressive and neurodegenerative condition associated with reduced levels of acetylcholine (Ach). In the brain, acetylcholine is primarily broken down by cholinesterase (ChE), making the inhibition of ChE an effective approach for treatment of AD. There are two types of ChE enzymes that exist in human nervous system: acetylcholinesterase (AChE) and butyrylcholinesterase (BChE). Currently, four anti- AChE agents—tacrine, rivastigmine, donepezil, and galantamine—are employed as drugs for Alzheimer’s disease (Hosen et al., 2023). However, their use is not without many severe side effects. Research findings suggest that as AD advances, there is an increase in the level and activity of butyrylcholinesterase (BChE), while conversely, the activity of acetylcholinesterase (AChE) decreases. Consequently, scientific research has shifted its focus towards the cholinergic system, particularly in the search for compounds that can inhibit AChE and BChE. Our ongoing research primarily centers on the identification of AChE (PDB ID: 4bdt) and BChE (PDB ID: 6qab) inhibitors using computer-based molecular docking method, leveraging phytochemicals derived from *V. diversifolium* plant.

**FIGURE 8:**
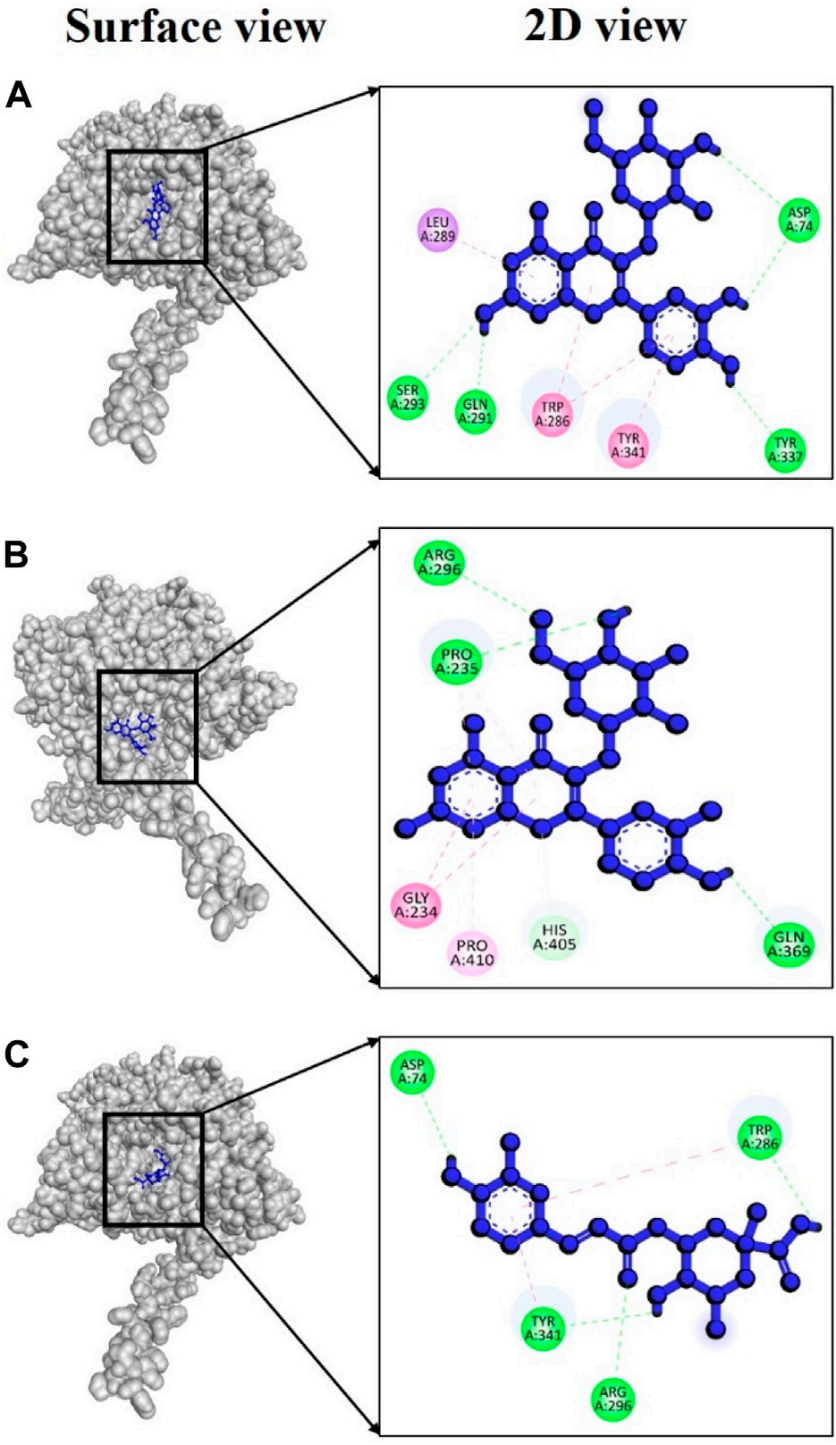
Docking interaction targeting AChE (PDB ID: 4bdt), where **(A)** Hyperoside, **(B)** Isoquercetin, **(C)** Neochlorogenic acid.

In the case of AChE, the complex 4bdt + hyperoside displayed a notably strong binding energy of −9.0 kcal/mol (Supplementary Table S6), indicating a highly dynamic interaction between these two molecules. Notably, within this complex, four hydrogen bonds were identified at amino acid residues Asp74, Gln291, Ser293, and Tyr337, with bond distances of 2.50, 1.85, 2.83, and 2.27Å (Figure 8A), respectively. In the case of the complex involving 4bdt and isoquercetin, a binding affinity of −8.5 kcal/mol (Supplementary Table S6) was observed, signifying a favorable interaction between these molecules. This complex featured three hydrogen bonds at Pro235 (2.68 Å), Arg296 (2.79 Å), and Gln369 (2.33 Å) residues (Figure 8B), underscoring the significance of their interaction. The complex formed by 4bdt and neochlorogenic acid exhibited a binding affinity of −8.4 kcal/mol (Supplementary Table S6). Within this complex, four hydrogen bonds were detected at Asp74, Trp286, Arg296, and Tyr341 residues, with bond distances of 2.44, 1.87, 3.01, and 2.63Å (Figure 8C), respectively, contributing to the overall stability of the complex.

**FIGURE 9:**
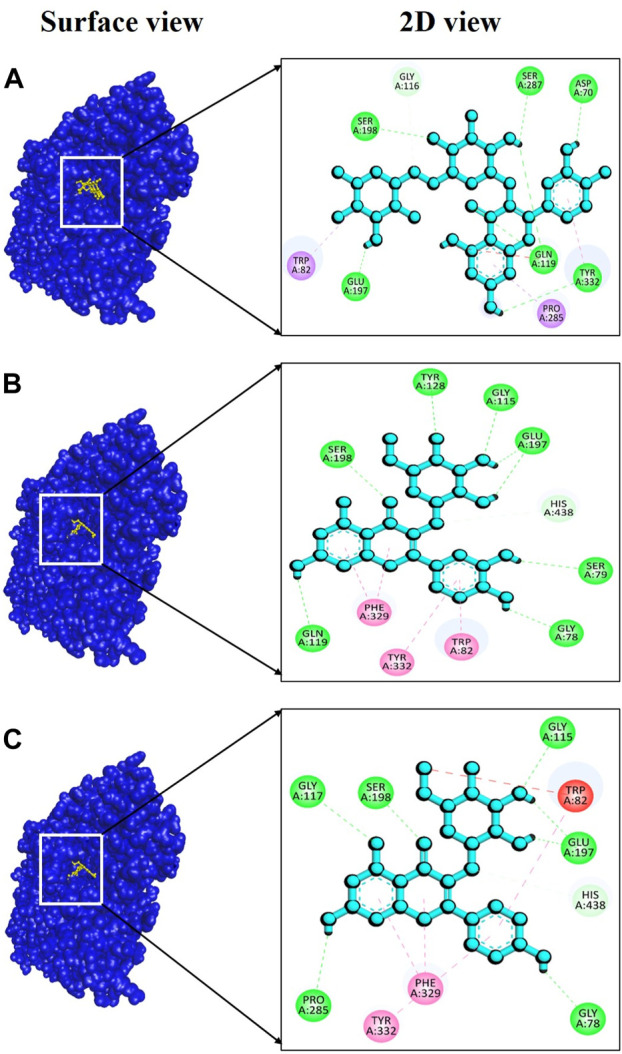
Docking interaction targeting BChE, (PDB ID: 6qab), where **(A)** Rutin, **(B)** Isoquercetin, **(C)** Neochlorogenic acid.

For BChE, the complex 6qab + rutin displayed a remarkably strong binding affinity, measured at −11.2 kcal/mol (Supplementary Table S7), indicating a highly favorable and stable interaction between the molecules involved. Notably, our analysis revealed the formation of six hydrogen bonds within this complex. These hydrogen bonds were established between specific amino acid residues involving Asp70, Gln119, Glu197, Ser198, Ser287 and Tyr332, with bond distances of 2.22, 2.42, 2.64, 2.53, 2.64, and 2.78 Å (Figure 9A), respectively, showcasing the complexity and strength of the molecular interaction. In the case of the complex involving 6qab + isoquercetin, a binding affinity of −10.8 kcal/mol (Supplementary Table S7) was observed. This complex featured seven hydrogen bonds at Gly78, Ser79, Gly115, Gln119, Tyr128, Glu197 and Ser198 with bond distances of 2.17, 2.89, 2.02, 3.00, 2.34, 2.29, and 2.03Å (Figure 9B), respectively, underscoring the significance of their interaction. The complex formed by 6qab + Neochlorogenic acid exhibited a binding affinity of −10.5 kcal/mol (Supplementary Table S7). Within this complex, six hydrogen bonds were detected at Gly78, Gly115, Gly117, Glu197, Ser198 and Pro285 residues, with bond distances of 2.27, 2.93, 2.93, 1.92, 2.03, and 2.85Å (Figure 9C), correspondingly.

## 4 Conclusion

To the best of our knowledge, this is the first report assessing the chemical compoundss, antioxidant, enzyme inhibitory potentials and cytotoxicity of *V. diversifolium*. Chemical profile and biological activities varied according to the type of extraction solvent and the results showed that *V. diversifolium* possessed compounds with interesting biological activities and consequently, it can be a potential source of nutraceutical, pharmacological and cosmetic ingredients. However, evaluation of the morpho-anatomical changes during plant growth and developments and their impact on the chemical composition and biological activity of the plant would be of interest. Also, further research on the isolation of bioactive molecules and illustration their mechanism of action as well as *in vivo* studies are recommended.

## Data Availability

Data available upon reasonable requests. Requests to access the datasets should be directed to: gokhanzengin@selcuk.edu.tr (GZ) and ali.eid@qu.edu.qa (AE).
