# Supplementary material for: Elucidating the chemical profile and biological studies of Verbascum diversifolium Hochst. extracts
Source: Front Pharmacol. 2024 Jan 30;15:1333865. doi: 10.3389/fphar.2024.1333865 (PMC10862011; doi:10.3389/fphar.2024.1333865)
Supplement: Supplementary file 1 [file Table1.docx]

**Table S1. The list of pathways where the cyclin dependent kinase 6 is involved identified by KEGG database.**

| Pathway identifier | Pathway name |
| --- | --- |
| hsa04110 | Cell cycle |
| hsa04115 | p53 signaling pathway |
| hsa04151 | PI3K-Akt signaling pathway |
| hsa04218 | Cellular senescence |
| hsa04934 | Cushing syndrome |
| hsa05160 | Hepatitis C |
| hsa05162 | Measles |
| hsa05163 | Human cytomegalovirus infection |
| hsa05164 | Influenza A |
| hsa05165 | Human papillomavirus infection |
| hsa05167 | Kaposi sarcoma-associated herpesvirus infection |
| hsa05169 | Epstein-Barr virus infection |
| hsa05200 | Pathways in cancer |
| hsa05203 | Viral carcinogenesis |
| hsa05206 | MicroRNAs in cancer |
| hsa05212 | Pancreatic cancer |
| hsa05214 | Glioma |
| hsa05218 | Melanoma |
| hsa05220 | Chronic myeloid leukemia |
| hsa05222 | Small cell lung cancer |
| hsa05223 | Non-small cell lung cancer |
| hsa05224 | Breast cancer |
| hsa05225 | Hepatocellular carcinoma |

**Table S2: Docking interaction targeting cyclin dependent kinase 6 (PDB ID: 1xo2)**

| Complex | Binding energy (kcal/mole) | Amino acid residues | Bond types |
| --- | --- | --- | --- |
| 1xo2+cyanidin-3- glucoside | -10.2 | Glu18  Ile19  Val101  Asp104  Thr107  Gln149  Leu152 | H  H  H  H  H  H  PA |
| 1xo2+Ellagic acid | -9.1 | Val101  Asp104  His100  Ala41  Ile19  Val27  Leu152 | H  H  CH  PA  PS  PS  PS |
| 1xo2+Fisetin (control) | -8.87 | Gln149  Thr185  Lys216  Pro217  Thr106 | H  H  CH  PA  PS |

*H: Hydrogen bond, CH: Conventional hydrogen bond, PA: Pi-alkyl bond, PS: Pi-sigma bond*

**Table S3: Docking interaction targeting tyrosinase (PDB ID: 3awu)**

| Complex | Binding energy (kcal/mole) | Amino acid residues | Bond types | Distance (Å) |
| --- | --- | --- | --- | --- |
| 3awu+Isoquercetin | -7.6 | Arg30  Asp69  Gln72  Trp254 | H  H  H  H | 1.25  3.04  2.84  2.59 |
| 3awu +Rutin | -7.5 | Arg30  Asp69  Phe34  Arg65 | H  H  PA  UDD | 2.74  2.57  4.67  1.34 |
| 3awu +Neochlorogenic acid | -7.3 | Arg30  Asp69  Trp254  Ala73 | H  H  H  PA | 2.63  2.46  2.86  4.89 |

*H: Hydrogen bond, PA: Pi-alkyl bond, UDD: Unfavourable donor-donor*

**Table S4: Docking interaction targeting alpha-amylase (PDB ID: 3baj)**

| Complex | Binding energy (kcal/mole) | Amino acid residues | Bond types | Distance (Å) |
| --- | --- | --- | --- | --- |
| 3baj+Rutin | -9.4 | Thr163  Glu233  His299  Asp300  His305  Trp59  Ile235  Try151  His201  Leu162  Lys200 | H  H  H  H  H  PS  PS  PP  PP  PA  UDD | 2.55  2.83  2.34  2.19  2.13  3.55  3.58  5.14  4.92  4.75  1.04 |
| 3baj+ Isoquercetin | -8.7 | Arg195  Glu233  His299  His305  Asp300  Ile235  Tyr151  His201  Leu162  Lys200  Asp197 | H  H  H  H  CH  PS  PP  PP  PA  PA  UDD | 2.85  2.67  2.60  2.76  3.06  3.71  4.88  5.78  5.82  5.19  2.61 |
| 3baj+Neochlorogenic acid | -7.9 | Gln63  Thr163  Asp187  Tyr62 | H  H  H  PP | 2.59  2.60  2.56  4.06 |

**Table S5: Docking interaction targeting alpha-glucosidase (PDB ID: 3w37)**

| Complex | Binding energy (kcal/mole) | Amino acid residues | Bond types | Distance (Å) |
| --- | --- | --- | --- | --- |
| 3w37+Rutin | -9.5 | Asp357  Met470  Ser474  Arg552  Phe476  Lys506  Asp232  Trp432 | H  H  H  H  PP  PA  PIA  UDD | 2.11  2.94  2.36  3.05  3.73  4.32  3.96  1.57 |
| 3w37+ kaempferol-3-glucoside | -7.4 | Glu105  Asn108  Arg113  Pro107  Arg102 | H  H  H  PA  PC | 2.19  2.30  2.55  4.79  4.14 |
| 3w37+Isoquercetin | -7.2 | Asp232  Asp568  Trp432  Phe476  Met470  Ala234  Lys506 | H  H  PP  PP  PS  UDD  UDD | 2.52  1.93  4.82  3.99  5.15  1.24  2.25 |

**Table S6: Docking interaction targeting AChE (PDB ID: 4bdt)**

| Complex | Binding energy (kcal/mole) | Amino acid residues | Bond types | Distance (Å) |
| --- | --- | --- | --- | --- |
| 4bdt +Hyperoside | -9.0 | Asp74  Gln291  Ser293  Tyr337  Leu289  Trp286  Tyr341 | H  H  H  H  PS  PP  PP | 2.50  1.85  2.83  2.27  3.56  5.55  3.89 |
| 4bdt+Isoquercetin | -8.5 | Pro235  Arg296  Gln369  His405  Pro410  Gly234 | H  H  H  CH  PA  AM | 2.68  2.79  2.33  3.28  4.64  5.28 |
| 4bdt+Neochlorogenic acid | -8.4 | Asp74  Trp286  Arg296  Tyr341 | H  H  H  H | 2.44  1.87  3.01  2.63 |

**Table S7: Docking interaction targeting BChE (PDB ID: 6qab)**

| Complex | Binding energy (kcal/mole) | Amino acid residues | Bond types | Distance (Å) |
| --- | --- | --- | --- | --- |
| 6qab +Rutin | -11.2 | Asp70  Gln119  Glu197  Ser198  Ser287  Tyr332  Gly116  Trp82  Pro285 | H  H  H  H  H  H  CH  PS  PS | 2.22  2.42  2.64  2.53  2.64  2.78  3.71  3.90  3.60 |
| 6qab +Isoquercetin | -10.8 | Gly78  Ser79  Gly115  Gln119  Tyr128  Glu197  Ser198  His438  Trp82  Phe329  Tyr332 | H  H  H  H  H  H  H  CH  PP  PP  PP | 2.17  2.89  2.02  3.00  2.34  2.29  2.03  3.52  4.79  4.90  5.11 |
| 6qab +Neochlorogenic acid | -10.5 | Gly78  Gly115  Gly117  Glu197  Ser198  Pro285  His438  Phe329  Tyr332  Trp82 | H  H  H  H  H  H  CH  PP  PP  UAA | 2.27  2.93  2.93  1.92  2.03  2..85  3.50  4.88  5.33  2.28 |
